# Prognostic Factors of MS Conversion in Optic Neuritis

**DOI:** 10.15844/pedneurbriefs-29-6-5

**Published:** 2015-06

**Authors:** Marytery Fajardo, Jennifer P. Rubin

**Affiliations:** 1 Division of Neurology, Ann & Robert H. Lurie Children's Hospital of Chicago, Chicago, IL; 2 Departments of Pediatrics and Neurology, Northwestern University Feinberg School of Medicine, Chicago, IL

**Keywords:** Optic Neuritis, Multiple Sclerosis, Oligoclonal Bands

## Abstract

Investigators from Children's Hospital Aschaffenburg, Germany; University of Manchester, Manchester, United Kingdom; University Children's Hospital Tübingen, Tübingen, Germany; and other international centers studied prognostic factors in optic neuritis.

Investigators from Children's Hospital Aschaffenburg, Germany; University of Manchester, Manchester, United Kingdom; University Children's Hospital Tübingen, Tübingen, Germany; and other international centers studied prognostic factors in optic neuritis. They retrospectively reviewed 357 children treated at 27 different hospitals presenting with isolated optic neuritis and with a median follow up of 4 years to evaluate for factors predicting eventual conversion to multiple sclerosis. Factors they reviewed included baseline abnormal MRI, presence of cerebrospinal fluid immunoglobulin G oligoclonal bands, sex, and laterality of optic neuritis (unilateral versus bilateral). They used Multiple Cox proportional-hazards regressions to identify abnormal MRI and CSF oligoclonal bands as independent predictors of conversion to multiple sclerosis, (cMRI: hazard ratio [HR]= 5.94, 95% confidence interval 3.39-10.39, p < 0.001; OCB: HR = 3.69, 95% CI = 2.32-5.86, p < 0.001), with an even higher risk when both factors were present (HR = 26.84, 95% CI = 12.26-58.74, p < 0.001). They also identified age as an independent risk factor, with a hazard ratio of 1.08 per year of age, 95% CI = 1.02-1.13, p = 0.003. They found that sex and whether the optic neuritis was unilateral or bilateral at presentation did not have any prognostic value. The authors also found that only 9 of 115 patients without oligoclonal bands and with a negative MRI developed MS within 4 years. Moreover, none of the 5 children with a final diagnosis of neuromyelitis optica had CSF oligoclonal bands during initial presentation. [1]

COMMENTARY. Although patients often improve significantly if not completely, optic neuritis in children often poses a prognostication challenge for the child neurologist. The authors of this study found a multiple sclerosis (MS) conversion rate of 40.6% after median of 4 years follow up, which is greater than prior studies have indicated, including 36% identified after two year follow up in a 2006 study [1, 2]. This finding may be related to more sensitive diagnostic criteria for MS than in past studies or a change in the natural history of childhood optic neuritis and MS [1, 3]. Regardless, this indicates that often, optic neuritis is not a benign self-limited event, and children who present with unilateral or bilateral optic neuritis may develop MS in the future.

Various studies have shown a benefit to prophylactic immunotherapy for adults with a clinically isolated syndrome [4], but similar data is not available for children [5]. It is then helpful for the clinician to have reliable prognostic evidence available to aid in decision making about when to initiate prophylactic treatment for MS. This study suggests that a thorough evaluation that includes CSF analysis for the presence of oligoclonal bands may be indicated in the initial evaluation of a pediatric patient presenting with optic neuritis, especially if MRI is not diagnostic of MS. Furthermore, this study reiterates the importance of following children with optic neuritis over time, with serial neuroimaging.
